# Structural Characterization of the Complex of SecB and Metallothionein-Labeled proOmpA by Cryo-Electron Microscopy

**DOI:** 10.1371/journal.pone.0047015

**Published:** 2012-10-04

**Authors:** Qiang Zhou, Shan Sun, Phang Tai, Sen-Fang Sui

**Affiliations:** 1 State Key Laboratory of Biomembrane and Membrane Biotechnology, Center for Structural Biology, School of Life Sciences, Tsinghua University, Beijing, China; 2 Department of Biology, Georgia State University, Atlanta, Georgia, United States of America; University of South Florida College of Medicine, United States of America

## Abstract

ProOmpA is a preprotein that is translocated across the plasma membrane by the general secretory pathway in *Escherichia coli*. The molecular chaperon SecB in Sec pathway can recognize and bind proOmpA for its translocation. However, the structure of the SecB/proOmpA complex remains unknown. Here, we constructed an uncleavable proOmpA fused with metallothionein at its C-terminus and labeled it with metals *in vitro* for the study of cryo-electron microscopy. Using single particle cryo-electron microscopy, we reconstructed 3D structure of the stable SecB/proOmpA complex. The structure shows that the major portion of preprotein locates on one side of SecB tetramer, resulting in an asymmetric binding pattern. This work also provides a possible approach to the structure determination of small protein complexes by cryo-electron microscopy.

## Introduction

The general secretory pathway (Sec pathway) is responsible for the translocation of a subset of proteins across the inner membrane to the periplasm in *E. coli*. SecB, a molecular chaperon in Sec pathway, recognizes and binds nascent preproteins with high affinity and low specificity [1], [2], [3], [4], [5], [6]. SecB maintains preproteins in a translocation-competent state by binding their mature regions [7], [8], [9], [10] and subsequently delivers them to the Sec translocase [11], [12], [13]. The interaction of SecB with preproteins has been studied thoroughly in kinetics [6], [14], [15], [16], [17]. However, the structure of the SecB/preprotein complex remains largely unclear, which limits our understanding of the molecular mechanism underlying how SecB interacts with preproteins.

ProOmpA, a model preprotein, is translocated across the cell membrane via Sec pathway in a post-translational manner in *E. coli* [18]. The signal peptide of proOmpA is cleaved by signal peptidase during the process of secretion, resulting in the formation of mature OmpA. This cleavage often makes the purified proOmpA contaminated with OmpA. In addition, the complex of SecB and proOmpA is very small with the molecular weight of only ∼110 kDa, and the complex tended to form aggregates *in vitro* [19]. All of these make the structural study of the SecB/proOmpA complex by cryo-electron microscopy (EM) difficult. In our previous work, only the 3D structure of the complex of SecB with OmpA was obtained by negative staining EM [19].

In this work, we performed two modifications in sample preparation to overcome the above problems. First, we constructed an uncleavable proOmpA by mutating its signal peptidase cleavage site from “AA” to “FP” [20], thus avoiding the contamination of mature OmpA. Second, we fused the uncleavable proOmpA to metallothionein (MT), a small cysteine-rich protein, to facilitate identification of its complex with SecB under cryo-EM. MT has been used as a genetic tag for cryo-EM in several studies [21], [22], [23], [24], [25]. Here, one or two copies of MT were added to the C-terminus of the uncleavable proOmpA and then labeled with Cd^2+^ or gold *in vitro*. Our results indicated that the uncleavable proOmpA-MT fusion protein (pOAMT) formed stable and uniform complex with SecB. We then used cryo-EM and single particle analysis to reconstruct the 3D structure of the complex at a resolution of ∼20 Å. The docking analysis of the crystal structure of SecB into the EM density map showed that the preprotein proOmpA binds to SecB asymmetrically.

## Materials and Methods

### Materials

SecB/OmpA complex [19], SecB [26], SecA [27], SecYEG-overexpressed inverted membrane vesicles (IMV) [28] were prepared as described previously.

### Plasmids Construction

**Figure 1:**
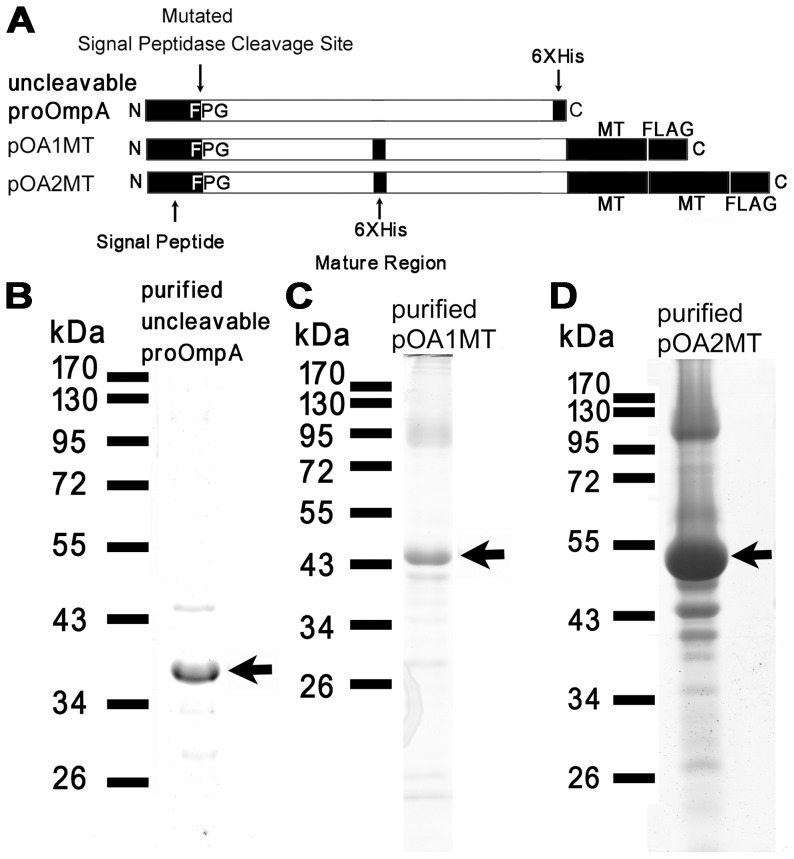
Cloning and purification of uncleavable proOmpA, pOA1MT and pOA2MT fusion proteins. (A) Schematic maps of uncleavable proOmpA, pOA1MT and pOA2MT constructs. The signal peptidase cleavage sites on these proteins were mutated to “FPG” from “AAP”. A His6 tag was attached to the C-terminus of uncleavable proOmpA or inserted into the middle of the mature region of pOA1MT and pOA2MT fusion proteins. A FLAG tag was added to the C-terminus of pOA1MT and pOA2MT. (B–D) The SDS-PAGE of purified uncleavable proOmpA (B), pOA1MT (C) and pOA2MT (D) stained with Coomassie brilliant blue R-250.

The uncleavable proOmpA was constructed into the *Nde*I-*Xho*I site of the pET21b vector. The mutation in the signal peptidase cleavage site from AAP to FPG (Fig. 1A) according to the previous report [20] was introduced by polymerase chain reaction (PCR)–based site-directed mutagenesis using the proOmpA expression vector [29] as a template. The obtained plasmid was sequenced and named pET21b-uncleavable proOmpA.

To construct the proOmpA-MT fusion proteins, the DNA fragment of uncleavable proOmpA was further subcloned into *Nde*I-*Bln*I site of the pET21b-1MT or pET21b-2MT [24]. In addition, a His_6_ tag was inserted between the 173th and 174th amino acid residues of uncleavable proOmpA by PCR–mediated site-directed mutagenesis (Fig. 1A). The obtained plasmids were sequenced and named pET21b-pOA1MT and pET21b-pOA2MT, respectively.

### Overexpression and Purification of Proteins

The pET21b-uncleavable proOmpA plasmid was transformed into *E. coli* BL21 (DE3) pLysS strains. The cells were cultured at 37°C, induced by adding isopropyl-β-D-thiogalactopyranoside to a final concentration of 1 mM when the OD_600_ of the cultures reached 0.6, and then incubated for additional 2 hours. Induced cells were collected by centrifugation, resuspended in 50 mM HEPES-KOH (pH 8.0), and then lysed by sonication. The lysate was centrifuged at 10,000 g for 10 minutes. The obtained pellet was extracted with 1.5% sarkosyl and dissolved in 50 mM Tris-HCl (pH 7.0) with 8 M urea. The dissolved uncleavable proOmpA protein was further centrifuged at 10,000 g for 10 minutes to remove insoluble materials.

The expression and purification of pOA1MT and pOA2MT fusion proteins were similar to that of uncleavable proOmpA protein except that the proteins were dissolved and stored in 50 mM HEPES-KOH (pH 7.5) with 50 mM KCl, 6 M guanine-HCl and 100 mM DTT.

### Metal Labeling of Fusion Proteins

The apo-state pOA1MT and pOA2MT were labeled with Cd^2+^ as described previously [30] with some modifications. Briefly, the purified apo-state fusion proteins were precipitated with 5 volumes of alcohol containing 100 mM DTT at −20°C for 30 minutes. The precipitated proteins were collected by centrifugation and dissolved in 10 mM acetic acid with 6 M guanine-HCl, and then dialyzed against the same buffer for 6 hours. The ultraviolet absorbance spectra of apo-state fusion proteins were measured after dialysis. For Cd^2+^ labeling, appropriate amount of CdCl_2_ in 0.1 M HCl was added into the fusion protein solutions. Then the solutions were dialyzed against 0.2 M ammonium acetate (pH 7.0) with 6 M guanine-HCl overnight and the free Cd^2+^ was removed by adding chelex-100 resin after dialysis. The Cd^2+^-bound fusion proteins were named pOA1MTCd and pOA2MTCd, respectively. To label gold, Cd^2+^-bound fusion proteins were diluted 20 folds into 25 mM Tris-HCl (pH 7.5) with 1% n-Dodecyl-β-D-maltoside (DDM) and 1 mM sodium aurothiomalate (International Laboratory, USA), and then incubated at 37°C for 3 hours [23]. The mixtures were centrifuged and the supernatants were precipitated with 5 volumes of alcohol at −20°C overnight. The precipitated proteins were collected by centrifugation and dissolved in 0.2 M ammonium acetate (pH 7.0) with 6 M guanine-HCl. The gold-bound fusion proteins were designated pOA1MTAu and pOA2MTAu, respectively. The numbers of bound gold atoms in gold-labeled fusion proteins were determined by dividing the value of the difference between the molecular mass of the gold-labeled and the apo-state fusion proteins by the gold atomic mass (197 Da).

### MALDI-TOF Mass Spectrometry

The Cd^2+^ or gold-labeled fusion proteins were analyzed on 4800 Plus MALDI-TOF/TOF mass spectrometer (Applied Biosystems). The fusion proteins were carefully desalted before the measurements. Spectra in mass range of 20,000–80,000 or 100,000 Da were collected.

### Formation and Purification of SecB/pOAMT Binary Complexes

The pOA1MTCd, pOA1MTAu and pOA2MTAu fusion proteins in 6 M guanine-HCl were rapidly diluted into 25 mM Tris-HCl (pH 7.5) containing SecB respectively. The mixtures were centrifuged at 12,000 rpm at room temperature for 10 minutes. The supernatants were subjected to Superdex-200 gel filtration chromatography and eluted with 25 mM Tris-HCl (pH 7.5). The eluted fractions were collected at 500 µl per tube and analyzed by SDS-PAGE. The first half of the elution peak was concentrated 10 folds to prepare cryo-EM sample.

### *In vitro* Translocation Assay of Preproteins

*In vitro* translocation of uncleavable proOmpA was assayed by the protease-accessibility method as previously described [28]. One modification was that the translocated uncleavable proOmpA was detected by western blotting with anti-His_6_ tag mouse monoclonal antibody. The method of *in vitro* translocation assay of pOAMT fusion proteins was similar to that of uncleavable proOmpA except that SecB and uncleavable proOmpA were substituted by the pre-prepared SecB/pOAMT complex in the translocation mixture, and the reaction was performed in the oxidative condition.

### Negative Stained EM and 3D Reconstruction of SecB/pOA1MTCd Complex

SecB/pOA1MTCd complex was applied to a glow-discharged continuous carbon-coated EM grid for 10 seconds and stained with 1% (w/v) uranyl acetate for 1 minute. The grid was examined under FEI Tecnai F20 at 200 kV. Image pairs were collected at tilt angles of 50° and 0° with Gatan UltraScan 4000 CCD at a nominal magnification of 80,000 times which was corresponding to 1.389 Å/pix. Initial model was generated by random conical tilt method using SPIDER and WEB [31]. 4696 particle pairs were selected from 66 image pairs using WEB and then binned by a factor of 2. The untilted particles were subjected to 2D classification. Eight initial models were created according to the classification. These models were similar to each other and one of them was chosen for further refine. The final 3D map was calculated from 15,935 untilted particles over eight cycles with the chosen initial model using EMAN [32]. The resolution of the map was estimated at about 24 Å by Fourier Shell Correlation (FSC). Surface representation was performed with UCSF Chimera [33]. The details of the processing are described in Text S1.

### Cryo-EM of SecB/OmpA, SecB/pOA1MTCd and SecB/pOA2MTAu Complex

The cryo-EM method for SecB/OmpA, SecB/pOA1MTCd and SecB/pOA2MTAu complex was the same as that for SecB/pOA1MTAu complex described below.

### Cryo-EM and Single Particle Analysis of SecB/pOA1MTAu Complex

SecB/pOA1MTAu complex was applied to glow-discharged holey carbon grid Quantifoil® R1.2/1.3 for 10 seconds. The grid was blotting for 1 second with filter paper and plunged into liquid nitrogen-cooled liquid ethane using Vitrobot®. The vitrified sample was transferred to FEI Tecnai F20 with Oxford CT3500 holder and images were collected at 200 kV at liquid nitrogen temperature under low-dose mode with electron dose of 20 e^−^/Å^2^ with defocus value of 1.5–3.0 µm using Gatan UltraScan 4000 CCD at a nominal magnification of 80,000 times which was corresponding to 1.389 Å/pix. 19562 complex particles were manually picked from 92 images, phase-flipped and then binned by a factor of 2 using EMAN [32]. The 2D-analysis of picked particles was performed with EMAN refine2d.py command. A cryo-EM 3D map of SecB/pOA1MTAu complex was reconstructed by refining the dataset at interval of 15° over eight cycles, using the negative stained 3D reconstruction of the SecB/pOA1MTCd complex as an initial model. The resolution of the map was estimated at about 20 Å by FSC. Surface representation and the docking of SecB crystal structure (PDB ID: 1QYN) was performed with UCSF Chimera [33]. The details of cryo-EM, image processing, and crystal structure docking are described in Text S1.

## Results

### Cloning and Purification of Uncleavable proOmpA, pOA1MT and pOA2MT

The schematic maps of uncleavable proOmpA, pOA1MT and pOA2MT were shown in Fig. 1A. The signal peptidase cleavage site on these preproteins was mutated from “AAP” to “FPG” to make them uncleavable. The His_6_ tag was added at different positions of the preproteins to detect the different translocation state in the *in vitro* translocation assay by western blotting. The C-terminal His_6_ tag was used to detect the full-translocation state, while the His_6_ tag in the middle of the mature region of pOA1MT and pOA2MT was used to detect the intermediate translocation state. The pOA1MT and pOA2MT contained one and two copies of MT for the metal labeling, respectively. As shown in Fig. 1B–D, any of the uncleavable proOmpA, pOA1MT, and pOA2MT proteins extracted from inclusion bodies showed one major band in SDS-PAGE analysis.

### Metal Labeling of pOA1MT and pOA2MT

**Figure 2:**
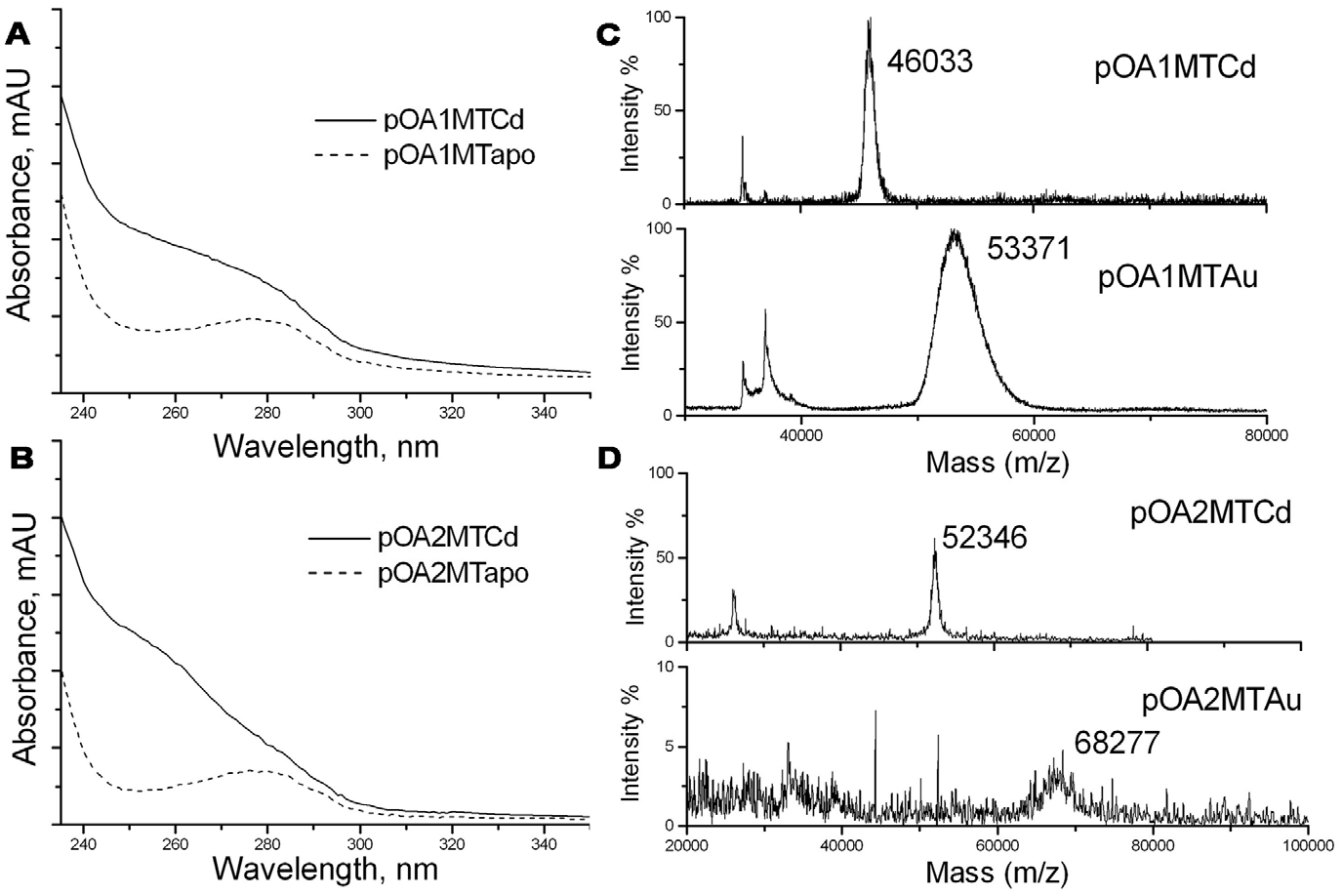
Metal labeling of the fusion proteins. (A, B) The absorbance spectra of pOA1MT (A) and pOA2MT (B) before (dot line) and after (solid line) labeling with Cd^2+^. (C) The MALDI-TOF mass spectra of the pOA1MTCd (upper panel) and pOA1MTAu (lower panel). (D) The MALDI-TOF mass spectra of the pOA2MTCd (upper panel) and pOA2MTAu (lower panel).

The ultraviolet absorbance spectrometry and MALDI-TOF mass spectrometry were carried out to characterize the metal binding ability of pOA1MT and pOA2MT proteins. As shown in Fig. 2, A and B, the ultraviolet absorbance spectra of either of the apo-state pOA1MT and pOA2MT fusion proteins exhibited one peak at 280 nm, a typical feature of pure protein, while that of either of the pOA1MTCd and pOA2MTCd proteins showed a prominent absorption from 240 nm to 300 nm, consistent with the previous reported MT-labeled protein [24]. These results suggested that the fusion proteins were labeled with Cd^2+^. Then the Cd^2+^ was replaced by gold to obtain the pOA1MTAu and pOA2MTAu proteins. All of the metal labeled proteins were subjected to MALDI-TOF mass spectrometry to analyze the mass change caused by metal labeling (Fig. 2, C and D). The measured mass of pOA1MTCd was 46,033 Da (Fig. 2C, upper panel), slightly larger than the nominal mass (45,725 Da) of the apo-state pOA1MT. When pOA1MTAu was measured, the mass peak was shifted to 53,371 Da (Fig. 2C, lower panel). This increment of the mass was equivalent to 39 gold atoms. Similarly, the measured mass of pOA2MTCd was 52,346 Da (Fig. 2D, upper panel), slightly larger than the nominal mass of the apo-state pOA2MT (52,192 Da). The measured mass of pOA2MTAu was 68,277 Da (Fig. 2D, lower panel). This increment of the mass was equivalent to 82 gold atoms. Thus, one copy of MT could bind about 40 gold atoms. A summary of gold-labeling results is shown in Table S1.

### *In vitro* Translocation Assay of Uncleavable proOmpA, pOA1MT and pOA2MT

**Figure 3:**
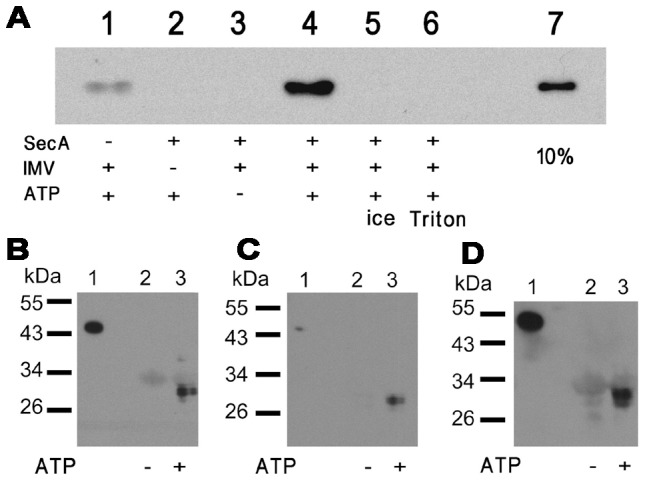
In vitro translocation assay of preproteins. (A) In vitro translocation assay of uncleavable proOmpA. ice, translocation reaction was performed on ice; Triton, 1% Triton X-100 was added during proteinase K digesting; 10%, 10% of total input of uncleavable proOmpA. (B–D) In vitro translocation assay of SecB/pOA1MTCd complex (B), SecB/pOA1MTAu complex (C), and SecB/pOA2MTAu complex (D). lane 1, the non-digested full-length precursor as the positive control; lane 2, absence of ATP; lane 3, presence of ATP.

We then investigated the translocation states of uncleavable proOmpA and metal-labeled pOAMT fusion proteins by *in vitro* translocation assay. As shown in Fig. 3A, the His_6_ tag at the C-terminus of proOmpA was detectable when SecA and ATP were added to initiate the reaction, indicating the whole of uncleavable proOmpA was translocated into the vesicles, thus protected from the protease degradation (lane 4). As controls, no protein was observed in the absence of IMV (lane 2) or ATP (lane 3), as well as when the reaction was performed on ice (lane 5), or the detergent was added to destroy the vesicles (lane 6). The presence of the weak proOmpA band without adding SecA in lane 1 may be due to the trace amount of SecA contained in IMV. The *in vitro* translocation assay of pOA1MT and pOA2MT fusion proteins was performed after their labeling with metal and using their complexes with SecB. A band corresponding to ∼30 kDa was present when the reaction mixture was probed with anti-His_6_ tag antibody for any of pOA1MTCd (Fig. 3B), pOA1MTAu (Fig. 3C) and pOA2MTAu (Fig. 3D), suggesting only a part of the fusion protein was protected, indicating the formation of the translocation intermediate.

### Formation and Purification of SecB/pOA1MTCd, SecB/pOA1MTAu and SecB/pOA2MTAu Complex

**Figure 4:**
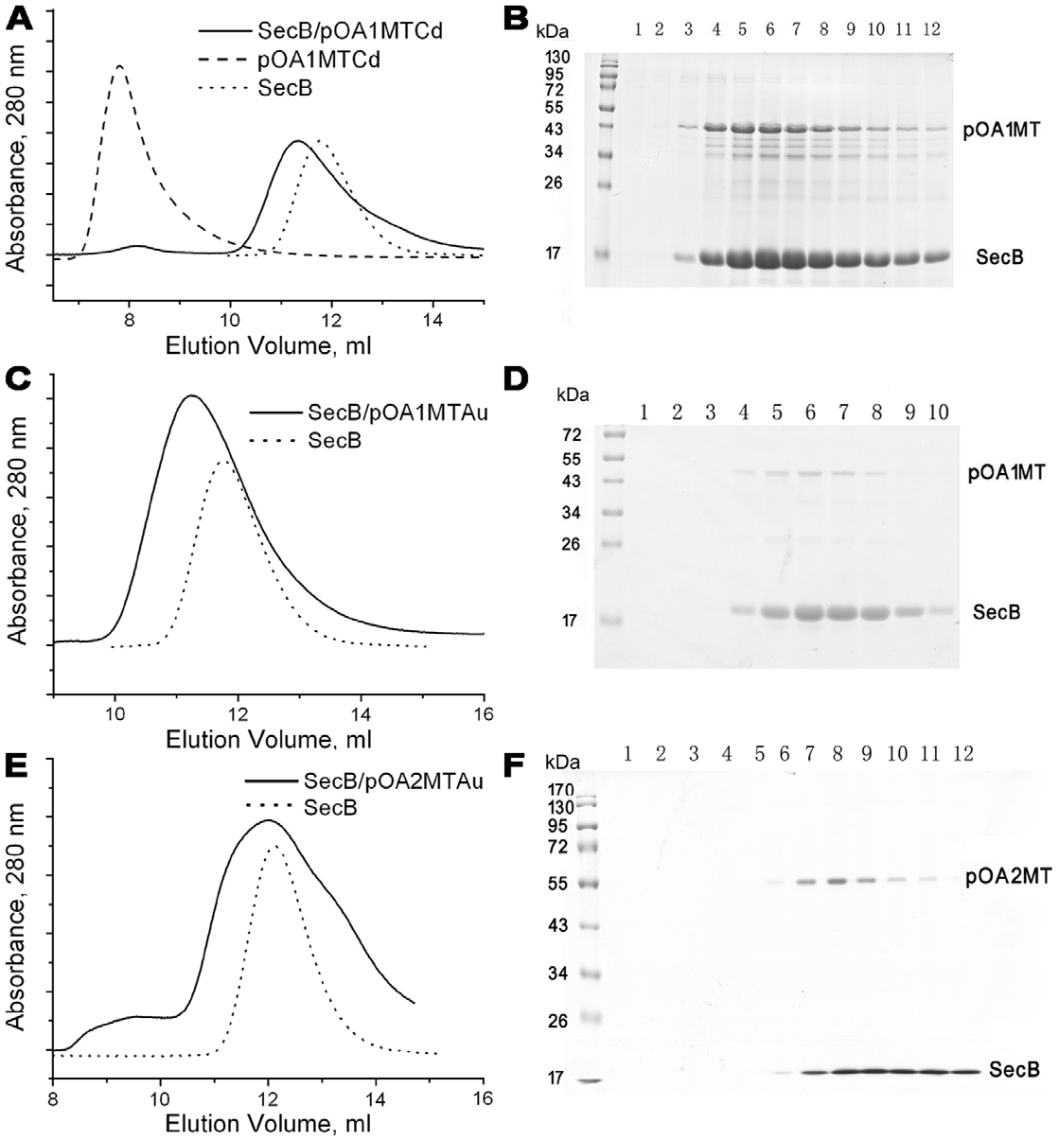
Gel filtration chromatography and SDS-PAGE analysis of SecB/pOAMT complexes. (A, C, E) Gel filtration analysis. Solid line, pOA1MTCd (A), pOA1MTAu (C), or pOA2MTAu (E) was diluted into a buffer with SecB; dash line, pOA1MTCd was diluted without SecB (A); dot line, SecB alone (A, C, E). (B, D, F) SDS-PAGE analysis. The eluted fractions of the SecB/pOA1MTCd complex from 9 ml to 15 ml (B), the SecB/pOA1MTAu complex from 8.5 ml to 13.5 ml (D), and the SecB/pOA2MTAu complex from 8 ml to 14 ml (F) were analyzed by SDS-PAGE with Coomassie brilliant blue R-250 staining.

The pOA1MTCd (Fig. 4A), pOA1MTAu (Fig. 4C) or pOA2MTAu (Fig. 4E) fusion protein was rapidly diluted into a buffer with or without SecB, and then the preparation was subjected to Superdex-200 gel filtration chromatography.

The pOA1MTCd alone was eluted at the void volume, indicating the formation of aggregates (Fig. 4A, dash line). When pOA1MTCd was diluted with SecB, a shift to a lower elution volume (11.2 ml, Fig. 4A, solid line) compared to that of SecB alone (11.7 ml, Fig. 4A, dot line) was observed, suggesting the formation of the complex, which was further confirmed by SDS-PAGE analysis of the eluted fractions from 9 ml to 15 ml. As shown in Fig. 4B, the major peak contained both SecB and pOA1MTCd, indicating the formation of the complex between these two proteins. Similar results were obtained with pOA1MTAu (Fig. 4, C and D) and pOA2MTAu (Fig. 4, E and F), except that pOA1MTAu or pOA2MTAu alone precipitated and was not examined by gel filtration. These results suggested that SecB could form complexes with pOA1MTCd, pOA1MTAu and pOA2MTAu, respectively.

### Visualization and 3D Reconstruction of SecB/pOA1MTAu Complex by Cryo-EM

**Figure 5:**
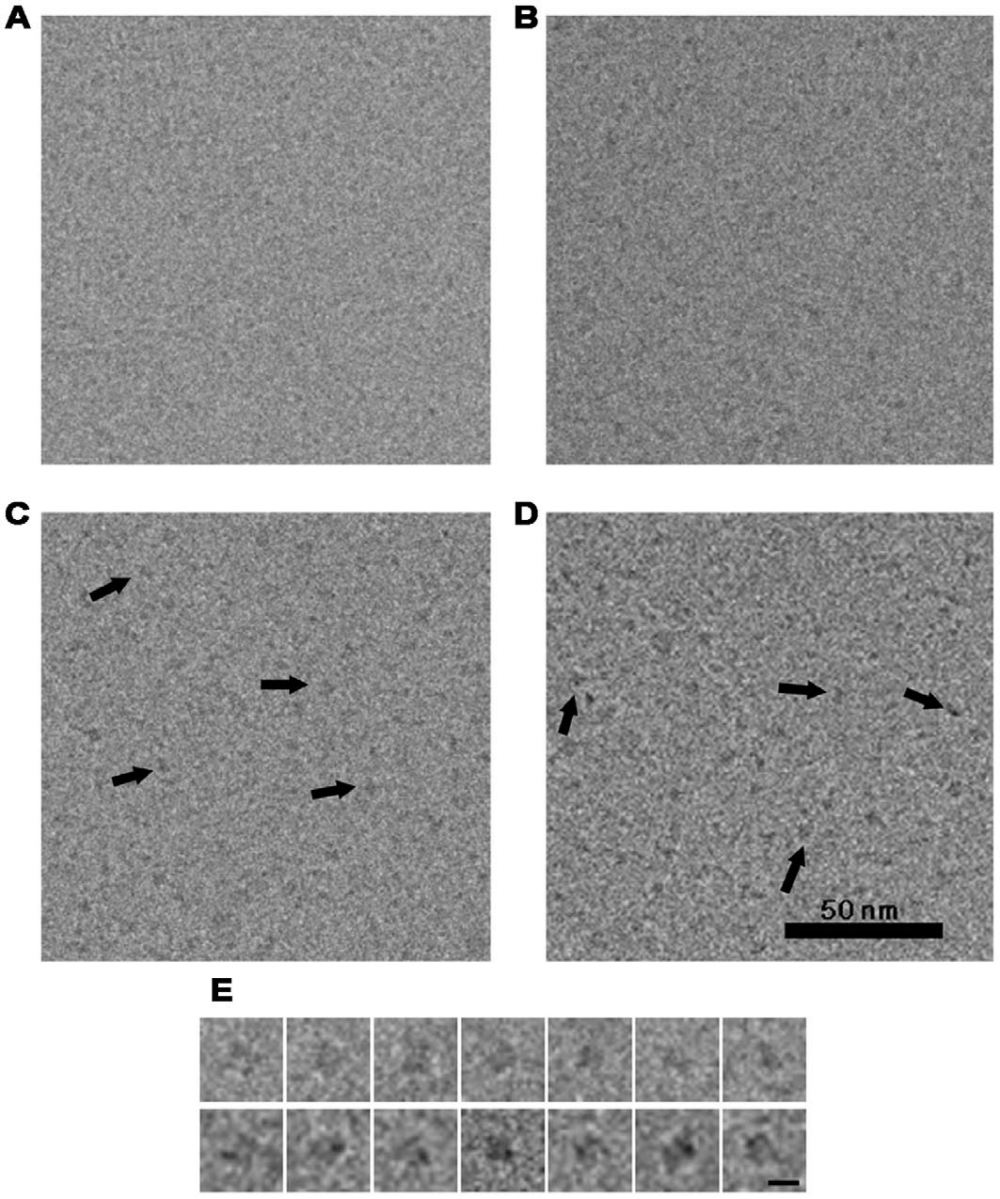
The comparison of cryo-EM images of different SecB/substrate complexes. (A–D) Cryo-EM images of SecB/OmpA complex (A), SecB/pOA1MTCd complex (B), SecB/pOA1MTAu complex (C), and SecB/pOA2MTAu complex (D). Several complex particles are indicated by arrows in (C) and (D). The defocus values for these images were about −1.85 µm. Scale bar, 50 nm. (E) Picked particles of the SecB/pOA1MTAu complex (upper panel) and the SecB/pOA2MTAu complex (lower panel). Scale bar, 5 nm.

The complexes, SecB/OmpA, SecB/pOA1MTCd, SecB/pOA1MTAu and SecB/pOA2MTAu, were imaged in vitreous ice with FEI Tecnai F20 (Fig. 5A–D). The complex particles of SecB/pOA1MTAu and SecB/pOA2MTAu could be observed clearly in the images and indicated by arrows (Fig. 5, C and D), though the defocus values of the images (∼1.85 µm) and the molecular weights of the complexes (∼120–130 kDa) were low. In contrast, the cryo-EM images of SecB/OmpA complex (Fig. 5A) and SecB/pOA1MTCd complex (Fig. 5B) acquired under similar conditions were not as visible as those of SecB/pOA1MTAu (Fig. 5C) and pOA2MTAu (Fig. 5D), indicating that labeling with gold indeed facilitated the identification of the small targets. Several particles of SecB/pOA1MTAu complex and SecB/pOA2MTAu complex were picked and displayed (Fig. 5E, upper panel and lower panel, respectively). It was notable that there were small black dots in only SecB/pOA2MTAu complex particles. These dots probably represented gold-bound MT.

**Figure 6:**
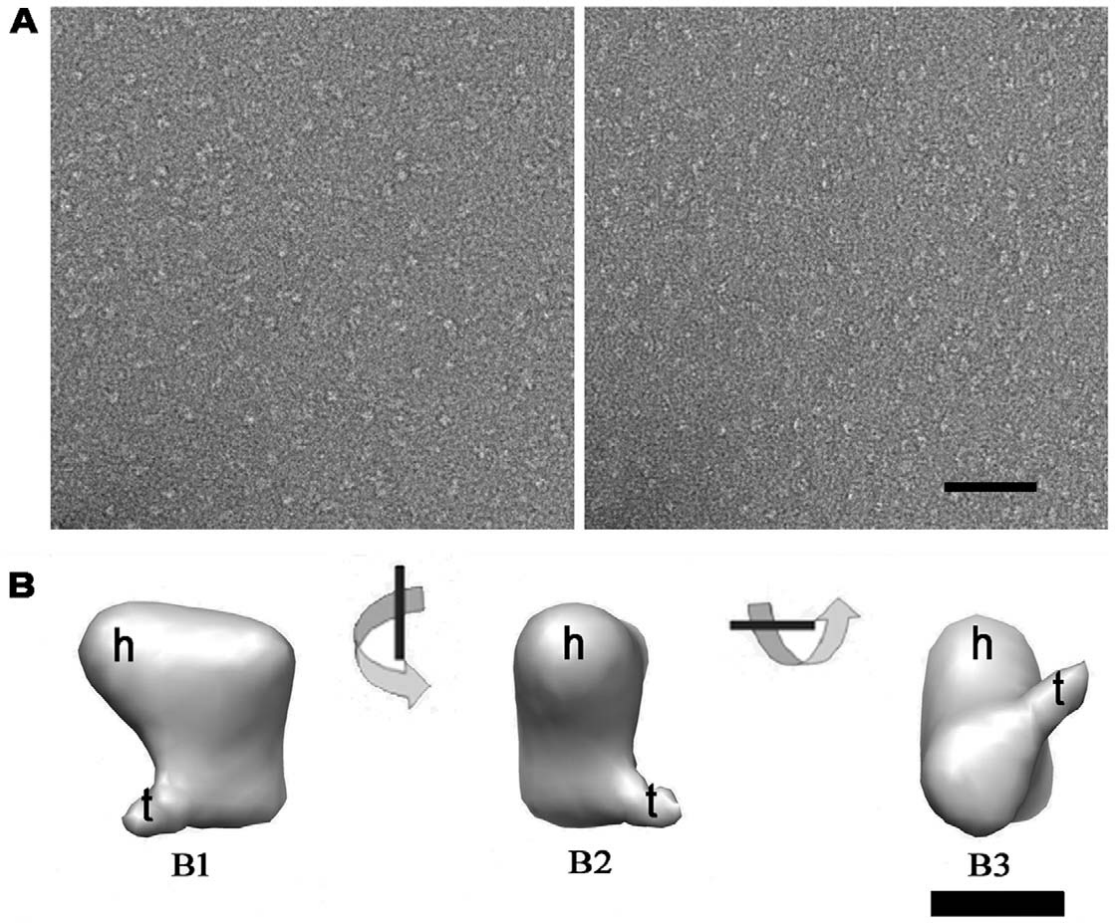
Negative staining EM 3D reconstruction of SecB/pOA1MTCd complex. (A) A tilt pair of SecB/pOA1MTCd complex. Left panel was 0°, right panel 50°. Scale bar, 50 nm. (B) Surface representation of the 3D reconstruction of SecB/pOA1MTCd complex. Three views were shown: top (B1), side (B2), and bottom (B3). These views related with each other by rotating 90° around the indicated axis. Letters h and t indicated the head and the tail of the 3D structure, respectively. Scale bar, 5 nm.

Next, we generated an initial structural model by negative staining EM with SecB/pOA1MTCd sample using random conical tilt method since the SecB/preprotein complexes were very small and lacking symmetry. The negative stained complex particles were homogeneous and evenly distributed (Fig. 6A, left panel). After collecting 66 tilt image pairs (Fig. 6A, left & right panel), a negatively stained 3D model at a resolution of about 24 Å (Fig. S1) was obtained and displayed at different views (Fig. 6B). The reconstruction 3D map had two protruding blobs. The bigger one was like a head, and the smaller one a tail, as indicated in Fig. 6B.

**Figure 7:**
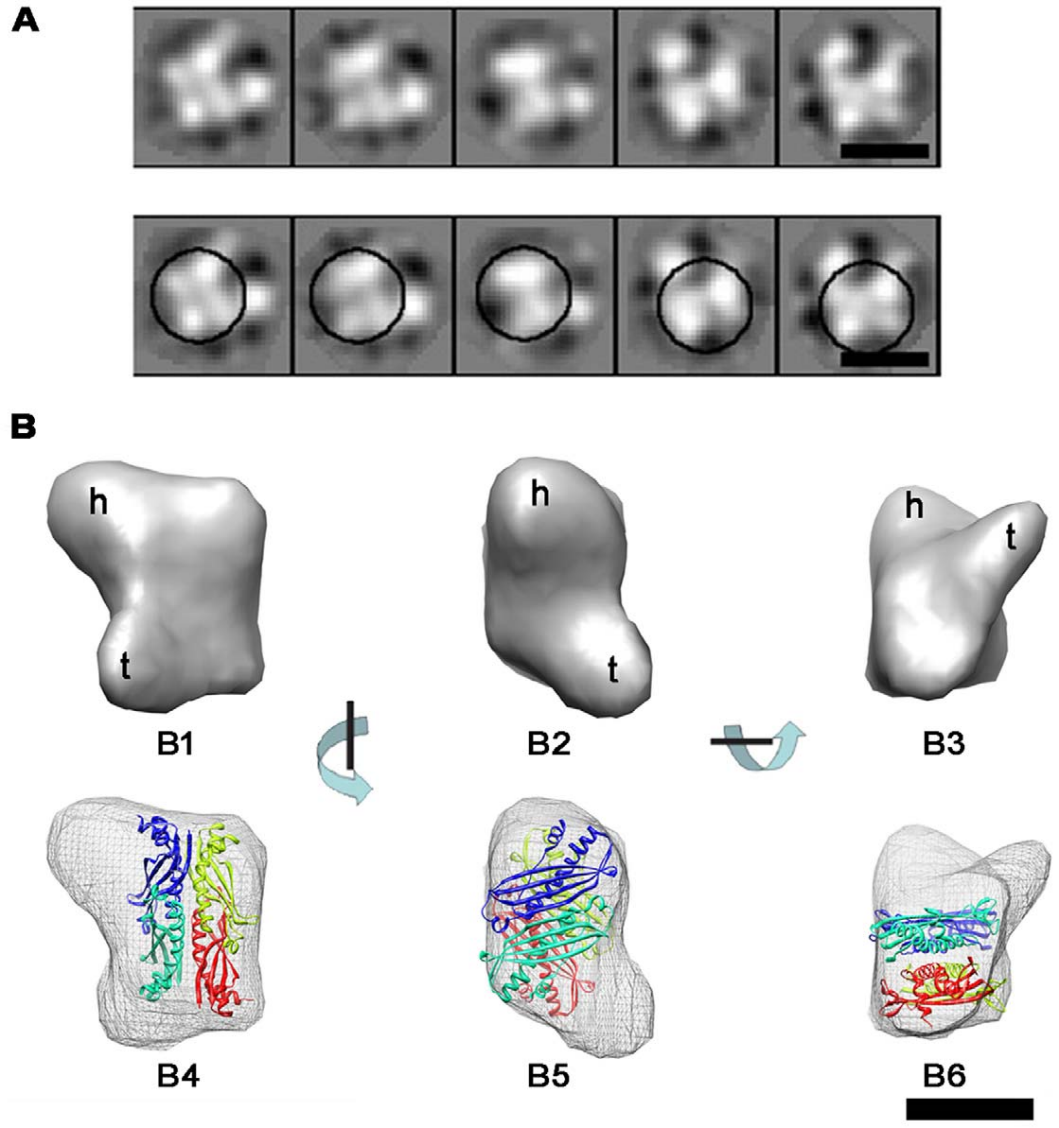
Cryo-EM 3D reconstruction of the SecB/pOA1MTAu complex. (A) Several 2D class averages of SecB/pOA1MTAu complex were shown in upper panel, and circles in the lower panel indicated SecB correspondingly. Scale bar, 5 nm. (B) Upper row, surface representation of cryo-EM 3D map. Three different views were shown: top (B1), side (B2), and bottom (B3). These views related with each other by rotating 90° around the indicated axis. Letters h and t indicated the head and the tail of the 3D structure, respectively. The lower row was shown for docking of the crystal structure of *E. coli* SecB (PDB ID: 1QYN) into the EM 3D map. B4–B6 were in the same orientations as B1-B3, respectively. The protomers of SecB were rendered in four colors: red, green, blue and cyanine. Scale bar, 5 nm.

Then, we performed 3D reconstruction of the SecB/pOA1MTAu complex by cryo-EM. Because the high contrast of MT moiety affected the alignment processing, the SecB/pOA2MTAu complex was not subjected to the single particle analysis. 19,562 SecB/pOA1MTAu complex particles were manually picked from 92 images and subjected to 2D analysis by EMAN refine2d.py command [32]. Several class averages were shown in the upper panel of Fig. 7A. SecB could be identified in these averages as indicated by circles in the lower panel of Fig. 7A correspondingly, and two additional densities were present around SecB. A 3D cryo-EM map at a resolution of about 20 Å (Fig. S2) was reconstructed by projection matching refinement against the initial model (Fig. 7B, upper row). To validate our cryo-EM 3D reconstruction, we did a tilt-test using a tilt-pair of the cryo-EM images of the SecB/pOA1MTAu complex (Text S1, Figure S3) by the program TILTDIFFMULTI [34]. As shown in the tilt-pair parameter plot (Figure S3), although there were some scattering, which may be due to the relatively low resolution, most of the particles were clustered around the preset titled angles. This should validate the cryo-EM map. The crystal structure (PDB ID: 1QYN) of *E. coli* SecB was manually docked into the map (Fig. 7B, lower row). In this docking, the main body was occupied by SecB with two protruding blobs, similar to the 3D map of the SecB/pOA1MTCd complex (Fig. 6B).

## Discussion

The interaction of preprotein with SecB is a crucial step for preprotein translocation across the plasma membrane. Here, we reconstructed the 3D structure of the complex of SecB with MT-labeled preprotein by cryo-EM for the first time. This work also shows the potential application of MT to cryo-EM of the small protein complex.

The proOmpA is used as a model substrate in protein translocation study for many years. However, the purified proOmpA is usually contaminated with mature OmpA. Some researcher used MM52, a temperature-sensitive *E. coli* strain, to express and purify proOmpA [35]. At a lethal temperature of 37°C, the mutated SecA in MM52 was defect to secrete preproteins, causing accumulation of proOmpA in the cytosol. But the pOAMT fusion proteins could not be expressed in MM52 in our experiments (data not shown). Another method to obtain pure proOmpA is to mutate its signal peptidase cleavage site, like uncleavable proOmpF-lpp [20]. Fortunately, unlike uncleavable proOmpF-lpp, which was very toxic to cells and failed to be overexpressed [20], the constructed uncleavable proOmpA was successfully expressed by using a tightly regulated expression system, pET21b vector and BL21 (DE3) pLysS cell.

We added one or two copies of MT to the C-terminus of uncleavable proOmpA. Three copies of MT were also performed but the fusion protein failed to be expressed. Our results indicated that only two copies of gold-bound MT, but not one copy, could be directly observed in cryo-EM images (Fig. 5E), suggesting that two copies of MT were not only sufficient but also necessary to be directly visible in cryo-EM images. This was in agreement with previous study [23]. It is notable that in our experiments, one copy of MT bound 39 gold atoms, and two copies of MT bound 82 gold atoms, thus about 40 gold atoms per copy of MT. This value was much greater than that reported previously, which was about 12–20 gold atoms [23]. The major difference in the gold labeling procedure was the addition of DDM into the reaction system in our work. DDM was a non-ionic detergent, and used to stabilize pOAMT fusion proteins. Although it is unknown whether the increased capability of gold labeling is due to the addition of DDM or not, which should be further examined using more samples, the findings here provided some useful information for further applying MT tag to cryo-EM research.

*In vitro* translocation assay showed pOA1MT or pOA2MT fusion protein formed translocation intermediate when it was translocated across the membrane (Fig. 3B–D). The intermediate was likely caused by the intra-molecular disulfide bridge, just as I31 [36], [37] and I29 [38], [39]. Thus, the pOA1MTAu or pOA2MTAu fusion protein could be a good candidate to study the translocase structure during the translocation process.

In previous work, the SecB/proOmpA complex formed aggregates *in vitro*, and were heterogeneous [19]. Here, all of the proOmpA-MT fusion proteins, pOA1MTCd, pOA1MTAu and pOA1MTAu, could form stoichiometric and homogeneous complexes with SecB, as evidenced by the gel filtration analysis and the EM images (Figs. 4, 5C and D, and 6A). Thus, the MT moiety may stabilize the preprotein during dilution and prevent it from aggregation.

As described in the introduction, SecB/proOmpA complex is very small with the molecular weight of only ∼110 kDa. Usually, protein complexes at this size are hard to be observed under cryo-EM. But in this work, with the use of gold labeled MT tag, we successfully identified the SecB/pOA1MTAu and SecB/pOA2MTAu in the vitreous ice. EM observation showed that all of SecB/pOA1MTCd (Fig. 6A), SecB/pOA1MTAu (Fig. 5C) and SecB/pOA2MTAu (Fig. 5D) complexes, were homogeneous and had characteristic features. In our previous 3D reconstruction of the SecB/OmpA complex by negative staining EM, there was an elongated density protruding from the main density [19]. Similarly, the 3D reconstruction of the SecB/pOA1MTCd or the SecB/pOA1MTAu complex in this work showed two blobs, one large like a head and one small like a tail, protruded from the main body (Fig. 6B and 7B). This was corresponding to the 2D class averages of the SecB/pOA1MTAu complex, in which two additional densities were present around SecB (Fig. 5F). Besides, it has been reported that SecB-bound substrate was in a near-native intermediate state containing secondary and tertiary structure [14], [40], [41]. Thus, the head and the tail may represent the tertiary structures in the substrate. Furthermore, multiple binding sites were suggested to exist on SecB [42], [43] and preprotein [7], [8], [9], [10]. Therefore, it is reasonable to deduce that the head and the tail located on the same side of SecB in the present structure suggested that the major portion of preprotein occupied only one side of SecB. This asymmetric binding pattern may lead to subsequent asymmetric interaction of SecB/preprotein complex with SecA [44], [45].

## Supporting Information

Figure S1 **Evaluation of 3D reconstruction of SecB/pOA1MTCd complex.** (A) Fourier Shell Correlation (FSC) curves of the 3D reconstruction. The calculated resolution was about 24 Å. (B) Angle distribution of the particles within asymmetrical triangle. The evenly distribution indicates that there was no missing region in Fourier space. (C) The comparison between model projections and class averages. The odd and even columns are model projections and class averages, respectively. The bar is 5 nm.
(TIF)

Figure S2 **Evaluation of 3D reconstruction of SecB/pOA1MTAu complex.** (A) FSC curves of the 3D reconstruction. The calculated resolution was about 20 Å. (B) Angle distribution of the particles within asymmetrical triangle. The evenly distribution indicates that there was no missing region in Fourier space. (C) The comparison between model projections and class averages. The odd and even columns are model projections and class averages, respectively. The bar is 5 nm.
(TIF)

Figure S3 **Tilt-test of the cryo-EM 3D reconstruction of the SecB/pOA1MTAu complex.** A, the tilt pair of the cryo-EM images of the SecB/pOA1MTAu complex. Left panel, 0°; right panel, 30°. Several particles are indicated by arrows. The bar represents 50 nm. B, The tilt-pair parameter plots of the tilted particle pairs. A red circle is centered at the preset tilt angle (30°). The red circle has a radius of 30° and includes ∼50% tilt-pairs of particles. The “+” symbols indicate that the particles have out-of-plane error larger than 1.5× the average.
(TIFF)

Table S1 **Molecular weight (MW) of pOAMT fusion proteins in different states and the numbers of gold atoms bound to gold-labeled fusion proteins.**
(DOC)

Text S1 **The detailed methods of negative staining EM, cryo-EM, image processing, the crystal structure docking, and the tilt-test of the 3D model.**
(DOC)
