# Correction: Structural Characterization of the Complex of SecB and Metallothionein-Labeled proOmpA by Cryo-Electron Microscopy

**DOI:** 10.1371/annotation/ee2d5478-c871-4c0e-859a-b6d4cde77a3a

**Published:** 2013-05-24

**Authors:** Qiang Zhou, Shan Sun, Phang Tai, Sen-Fang Sui

There was a name missing from the Acknowledgments. The following is the correct version:

We thank A. Miyazawa, A. J. Driessen and D. Oliver for providing the strains and the plasmids. We thank R.Henderson for providing the programs TILTDIFF and TILTDIFFMULTI. We also thank the comments of H.W. Wang. 

