# Supplementary figures and images for: Structural Characterization of the Complex of SecB and Metallothionein-Labeled proOmpA by Cryo-Electron Microscopy
Source: PLoS One. 2012 Oct 4;7(10):e47015. doi: 10.1371/journal.pone.0047015 (PMC3464278; doi:10.1371/journal.pone.0047015)

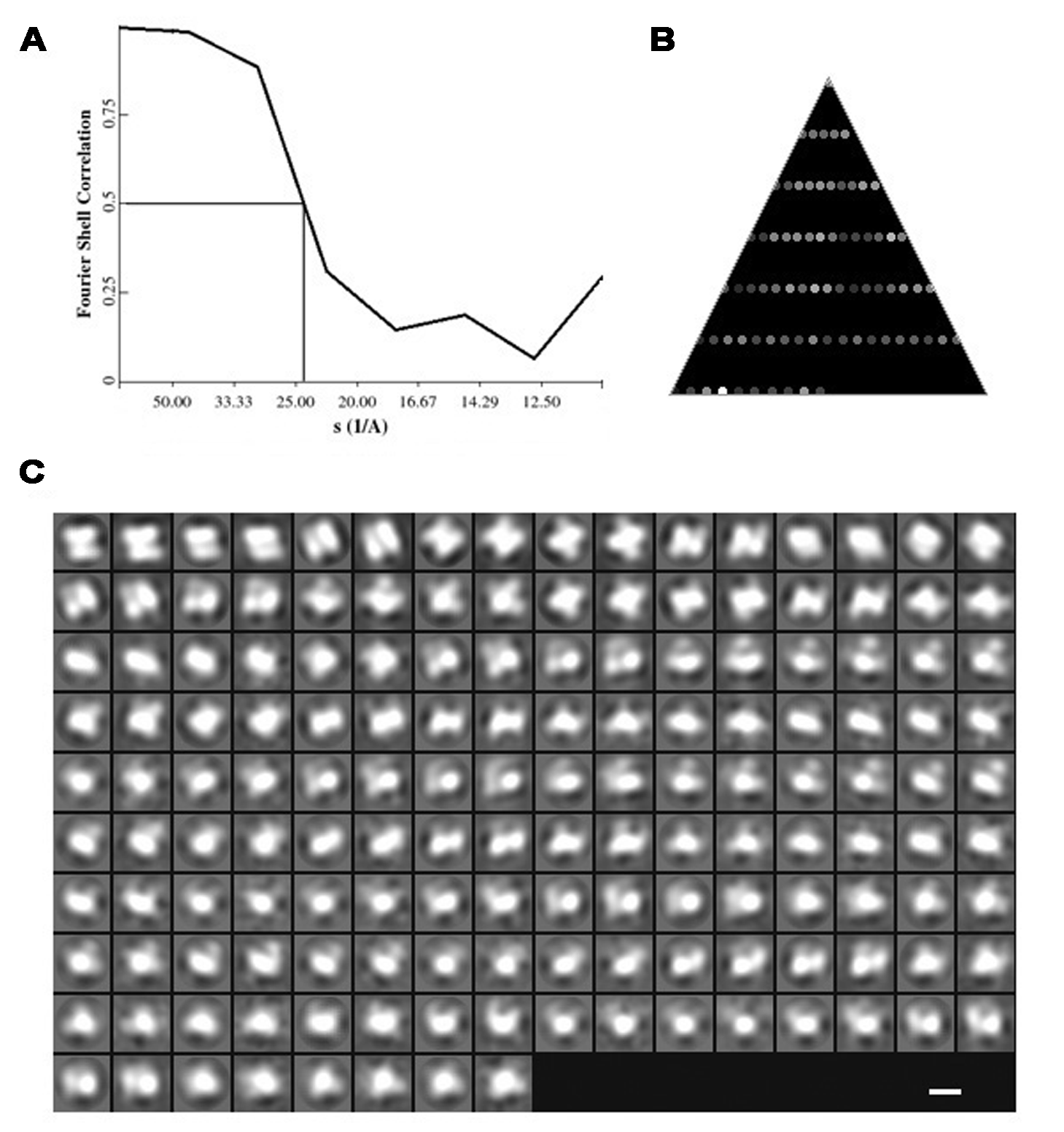

Supplement: Figure S1 — Evaluation of 3D reconstruction of SecB/pOA1MTCd complex. (A) Fourier Shell Correlation (FSC) curves of the 3D reconstruction. The calculated resolution was about 24 Å. (B) Angle distribution of the particles within asymmetrical triangle. The evenly distribution indicates that there was no missing region in Fourier space. (C) The comparison between model projections and class averages. The odd and even columns are model projections and class averages, respectively. The bar is 5 nm. (TIF) [file pone.0047015.s001.tif]

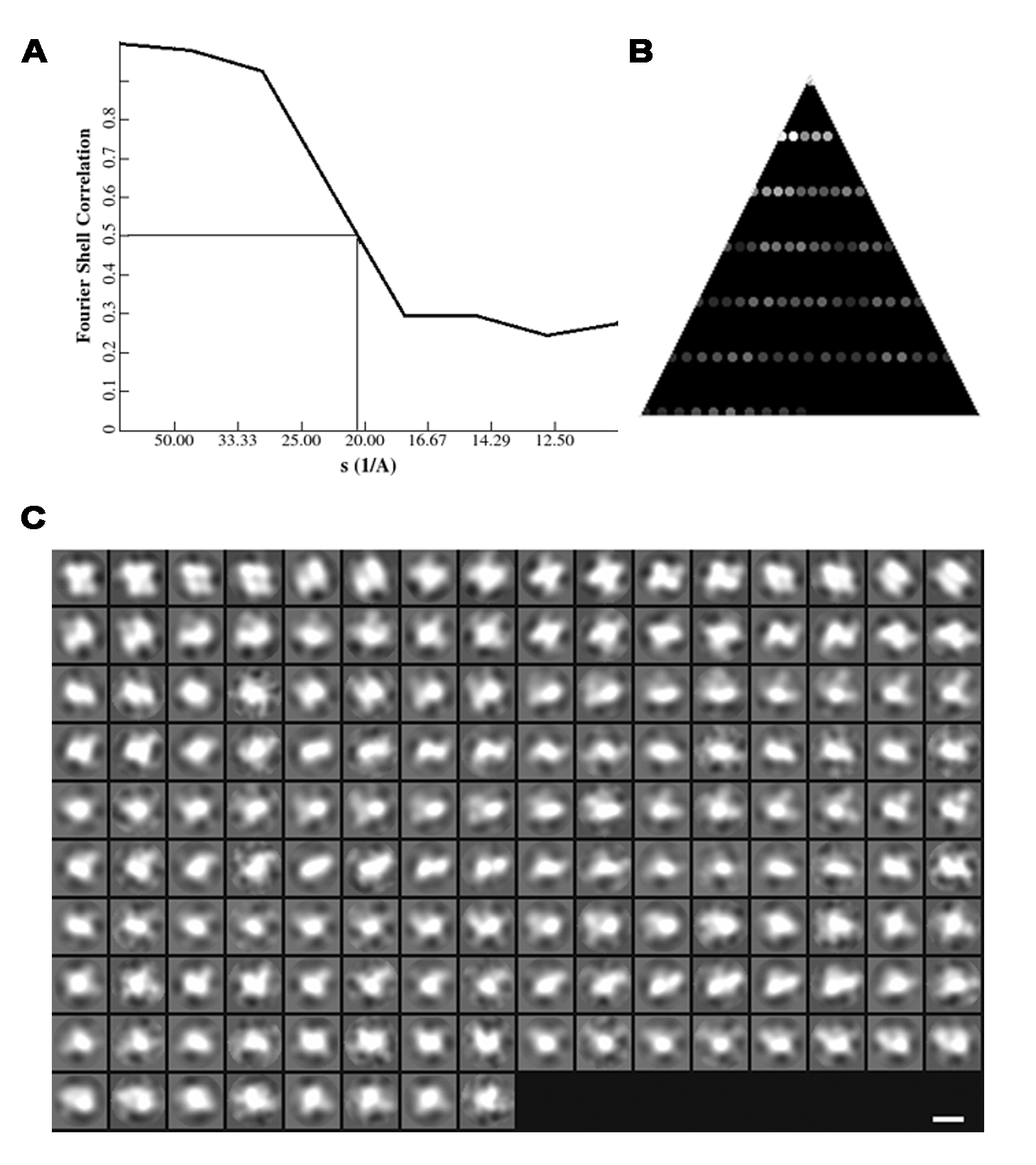

Supplement: Figure S2 — Evaluation of 3D reconstruction of SecB/pOA1MTAu complex. (A) FSC curves of the 3D reconstruction. The calculated resolution was about 20 Å. (B) Angle distribution of the particles within asymmetrical triangle. The evenly distribution indicates that there was no missing region in Fourier space. (C) The comparison between model projections and class averages. The odd and even columns are model projections and class averages, respectively. The bar is 5 nm. (TIF) [file pone.0047015.s002.tif]

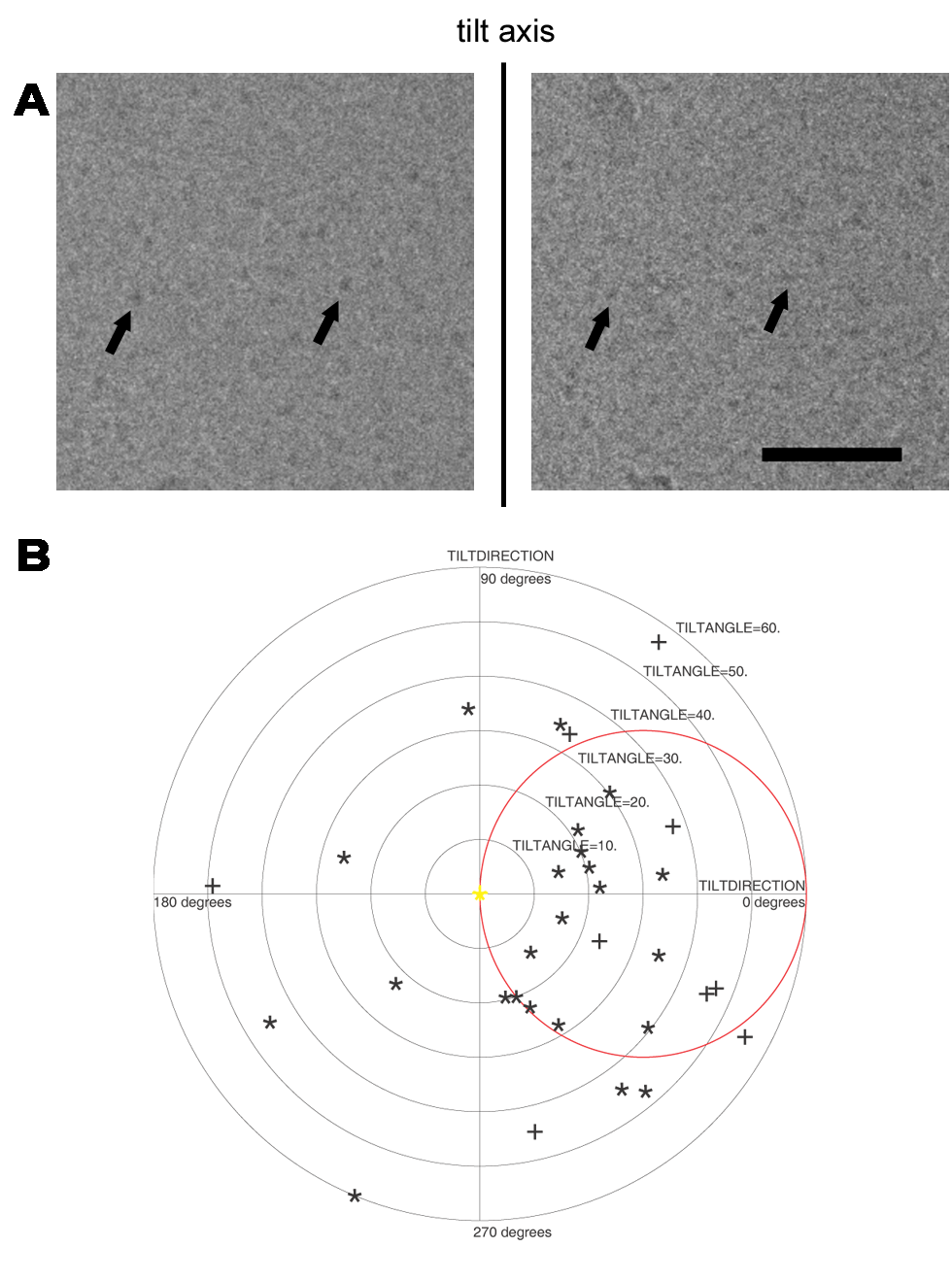

Supplement: Figure S3 — Tilt-test of the cryo-EM 3D reconstruction of the SecB/pOA1MTAu complex. A, the tilt pair of the cryo-EM images of the SecB/pOA1MTAu complex. Left panel, 0°; right panel, 30°. Several particles are indicated by arrows. The bar represents 50 nm. B, The tilt-pair parameter plots of the tilted particle pairs. A red circle is centered at the preset tilt angle (30°). The red circle has a radius of 30° and includes ∼50% tilt-pairs of particles. The “+” symbols indicate that the particles have out-of-plane error larger than 1.5× the average. (TIFF) [file pone.0047015.s003.tif]
