# Supplementary material for: Structural Characterization of the Complex of SecB and Metallothionein-Labeled proOmpA by Cryo-Electron Microscopy
Source: PLoS One. 2012 Oct 4;7(10):e47015. doi: 10.1371/journal.pone.0047015 (PMC3464278; doi:10.1371/journal.pone.0047015)
Supplement: Table S1 — Molecular weight (MW) of pOAMT fusion proteins in different states and the numbers of gold atoms bound to gold-labeled fusion proteins. (DOC) [file pone.0047015.s004.doc]

| Fusion protein | MW_apo-state_ (Da) | MW_Cd2+-labeled_ (Da) | MW_Gold-labeled_ (Da) | Number of bound gold atoms* |
| --- | --- | --- | --- | --- |
| pOA1MT | 45,725 | 46,033 | 53,371 | 39 |
| pOA2MT | 52,192 | 52,346 | 68,277 | 82 |

Table S1. Molecular weight (MW) of pOAMT fusion proteins in different states and the numbers of gold atoms bound to gold-labeled fusion proteins.

* The number of gold atoms bound (*N*_gold_) to gold-labeled fusion protein is calculated by the formula : *N*_gold_= (*MW*_gold-labeled_-*MW*_apo-state_)/*M*_gold_, where *M*_gold_=197 Da.
