# Supplementary material for: Structural Characterization of the Complex of SecB and Metallothionein-Labeled proOmpA by Cryo-Electron Microscopy
Source: PLoS One. 2012 Oct 4;7(10):e47015. doi: 10.1371/journal.pone.0047015 (PMC3464278; doi:10.1371/journal.pone.0047015)
Supplement: Text S1 — The detailed methods of negative staining EM, cryo-EM, image processing, the crystal structure docking, and the tilt-test of the 3D model. (DOC) [file pone.0047015.s005.doc]

**Supporting Information Text S1**

**Negative stained EM and 3D reconstruction of SecB/pOA1MTCd complex**

SecB/pOA1MTCd complex was applied to a glow-discharged continuous carbon-coated EM grid for 10 seconds and stained with 1% (w/v) uranyl acetate for 1 minute. The grid was examined under FEI Tecnai F20 at 200 kV. Image pairs were collected at tilt angles of 50° and 0° with Gatan UltraScan 4000 CCD at a nominal magnification of 80,000 times which was corresponding to 1.389 Å/pix. Initial model was generated by random conical tilt method using SPIDER and WEB [1]. 4696 particle pairs were selected from 66 image pairs using WEB and then binned by a factor of 2. The untilted particles were subjected to reference-free alignment using “AP SR” command of SPIDER. The reference-free aligned particles was classified into eight classes by Hierarchy method. One 3D models were obtained from each class according to the standard protocols of random conical reconstruction. There were eight 3D models obtained in total. These models were similar to each other and one of them was used as the initial model of the final refinement. For the final refinement, 15,935 untilted particles were picked out from untilted images using BOXER program in EMAN [2]. The picked particles were phase flipped and binned by a factor of 2 before further processing. The projection-matching refinement was performed at 15° interval for eight cycles with the data set of 15,935 untilted particles using EMAN. The final 3D map was calculated with 13,342 particles. The resolution of the final 3D map was estimated at about 24 Å by Fourier Shell Correlation (FSC). Surface representation was performed with UCSF Chimera [3] and the threshold was selected to accommodate 115 kDa of mass.

**Cryo-EM and single particle analysis of SecB/pOA1MTAu complex**

SecB/pOA1MTAu complex was applied to glow-discharged holey carbon grid Quantifoil® R1.2/1.3 for 10 seconds. The grid was blotting for 1 second with filter paper and plunged into liquid nitrogen-cooled liquid ethane using Vitrobot®. The vitrified sample was transferred to FEI Tecnai F20 with Oxford CT3500 holder and images were collected at 200 kV at liquid nitrogen temperature under low-dose mode with electron dose of 20 e^-^/Å^2^ with defocus value of 1.5-3.0 μm using Gatan UltraScan 4000 CCD at a nominal magnification of 80,000 times which was corresponding to 1.389 Å/pix. 19562 complex particles were manually picked from 92 images with BOXER program in EMAN [2], The picked particles were phase-flipped and binned by a factor of 2 before further processing. For 2D analysis, the particles was classified into 100 classes using refine2d.py command of EMAN. For 3D reconstruction, the 3D structure of the negative stained SecB/pOA1MTCd complex was used as an initial model. The projection-matching refinement was performed at 15° interval for eight cycles with the dataset of 19562 complex particles using EMAN. The final 3D map was calculated with 10,867 particles. The resolution of the map was estimated at about 20 Å by FSC. Surface representation was performed with UCSF Chimera [3] and the threshold was selected to accommodate 122 kDa of mass.

**The docking of the crystal structure**

The docking of the crystal structure of SecB (PDB ID: 1QYN) into the 3D map was performed using the “Fit model in map” in UCSF Chimera [3]. The position of SecB in the map was determined to consistent with the 2D class average shown in Figure 7A. The orientation of SecB was further determined by automatic fitting. The correlation coefficient for the docking of the crystal structure of SecB into the 3D map is 0.9117.

**Tilt-test of the cryo-EM 3D reconstruction of the SecB/pOA1MTAu complex**

A tilt-pair of cryo-EM images of the SecB/pOA1MTAu complex was recorded at 0° and 30° under the same condition as described above other than the electron dose was reduced to 15 e^-^/Å^2^ for each exposure. 32 tilt-pairs of particles were picked out with WEB [1]. The Euler angles of the tilted and untilted particles were determined by comparing them with the cryo-EM 3D model of the SecB/pOA1MTAu complex using SPIDER [1]. The tilt-pair parameter plot of these particles was produced using the program TILTDIFFMULTI [4]. A red circle is centered at the preset tilt angle (30°). The red circle has a radius of 30° and includes ~50% of tested tilt-pairs of particles. The particles with out-of-plane error larger than 1.5x average are indicated by “+” symbols.

**References**

1 Frank J, Radermacher M, Penczek P, Zhu J, Li Y, et al. (1996) SPIDER and WEB: processing and visualization of images in 3D electron microscopy and related fields. J Struct Biol 116: 190-199

2 Ludtke SJ, Baldwin PR, Chiu W (1999) EMAN: Semiautomated software for high-resolution single-particle reconstructions. J Struct Biol 128: 82-97

3 Pettersen EF, Goddard TD, Huang CC, Couch GS, Greenblatt DM, et al. (2004) UCSF Chimera--a visualization system for exploratory research and analysis. J Comput Chem 25: 1605-1612

4 Henderson R, Chen SX, Chen JZ, Grigorieff N, Passmore LA, et al. (2011) Tilt-Pair Analysis of Images from a Range of Different Specimens in Single-Particle Electron Cryomicroscopy. J Mol Biol 413:1028–1046
